# Effectiveness and moderators of individual cognitive behavioral therapy versus treatment as usual in clinically depressed adolescents: a randomized controlled trial

**DOI:** 10.1038/s41598-020-71160-1

**Published:** 2020-09-09

**Authors:** Yvonne Stikkelbroek, Gerko Vink, Maaike H. Nauta, Marco A. Bottelier, Leonieke J. J. Vet, Cathelijne M. Lont, Anneloes L. van Baar, Denise H. M. Bodden

**Affiliations:** 1 Child and Adolescent Studies, Utrecht University, PO Box 80.140, 3508 TC Utrecht, The Netherlands; 2 Faculty of Behavioral and Social Sciences, University of Groningen, Groningen, The Netherlands; 3 Center for Child and Youth Psychiatry, Triversum, The Netherlands; 4 Child Study Center, Accare, The Netherlands; 5 Child and Youth Psychiatry, Altrecht, Utrecht, The Netherlands

**Keywords:** Depression, Paediatrics

## Abstract

We examined if manualized cognitive behavioral therapy (CBT) was more effective than Treatment As Usual (TAU) for clinically depressed adolescents within routine care. This multisite Randomized controlled trail included 88 clinically depressed adolescents (aged 12–21 years) randomly assigned to CBT or TAU. Multiple assessments (pre-, post treatment and six-month follow-up) were done using semi-structured interviews, questionnaires and ratings and multiple informants. The primary outcome was depressive or dysthymic disorder based on the KSADS. Completers, CBT (n = 19) and TAU (n = 26), showed a significant reduction of affective diagnoses at post treatment (76% versus 76%) and after six months (90% versus 79%). Intention-to-treat analyses on depressive symptoms showed that 41.6% within CBT and 31.8% within the TAU condition was below clinical cut-off at post treatment and after six-months, respectively 61.4% and 47.7%. No significant differences in self-reported depressive symptoms between CBT and TAU were found. No prediction or moderation effects were found for age, gender, child/parent educational level, suicidal criteria, comorbidity, and severity of depression. We conclude that CBT did not outperform TAU in clinical practice in the Netherlands. Both treatments were found to be suitable to treat clinically referred depressed adolescents. CBT needs further improvement to decrease symptom levels below the clinical cut-off at post treatment.

## Introduction

Depression is identified as a rapidly growing epidemic disease that will be the leading cause of the global burden of disease in 2030^1^. Even more worrisome, the highest incidence rate of depression is found during adolescence^2,3^. Depression is also the predominant cause of illness and disability for youth aged 10 to 19 years^1^. Depressive disorders are a serious threat for adolescent mental health because of the high prevalence rate^4–7^, high burden of disease^8^ and high rate of recurrence^9,10^. In addition, depressed adolescents showed an increased risk of suicide, as well as for social problems, delinquency, learning problems, substance abuse problems, physical problems, teen pregnancies and experiencing negative life events^10–12^. Therefore, it is important that depressive disorders are treated effectively at an early stage^10,13^.

Cognitive behavior therapy (CBT) and Interpersonal therapy (IPT) are identified as the psychological treatments of choice besides medication^14,15^. Different meta-analyses on the effectiveness of a broad range of psychological interventions for adolescent depression showed effect sizes ranging from small-to-moderate (0.34)^16^ to large (1.27)^17^. In a meta-analysis solely directed at the effectiveness of CBT in depressed youth, a medium effect size of 0.53 was found^18^. A review of Watanabe and colleagues^19^ showed that 50% of the adolescents did not meet criteria of a depression diagnosis after CBT compared to 35% in the TAU condition. Furthermore, a network meta-analysis (a novel approach that integrates direct and indirect evidence from randomized controlled studies) showed that CBT was significantly more effective than control conditions in treating depression in youth (including placebo, waitlist, and treatment-as-usual)^20^. However, most studies included in these meta-analyses are efficacy studies, conducted in “ideal” and controlled circumstances. Such studies lack generalizability to “real-world” clinical practice, because their samples do not match the complex and severe cases seen in routine mental health care^16^.

Although CBT is one of the most intensively studied psychological treatment of which efficacy is repeatedly established against a broad range of control conditions, it is still unclear if CBT can outperform an active treatment condition within routine mental health care. It is suggested that highly structured and manualized CBT can be beneficial when confronted with complex client conditions^21^. However, the effectiveness of CBT in clinically depressed adolescents referred to routine mental health care still needs to be established.

For this study, the CBT program “Coping with Depression course for Adolescents (CWD-A)”^22^ designed for groups, was translated into Dutch and adapted to an individual version, the “D(o)epression course”^23,24^. Several RCT’s have repeatedly shown that CWD-A is more effective than inactive control conditions^25–31^ and treatment as usual^26^, However, these results are also inconclusive^32^. When comparing the results of several studies on CWD-A with an active control condition (which consisted of Care as usual, Interpersonal therapy or CWD-A plus a parent training) for adolescents diagnosed with a depression, a small-to-moderate effect size of 0.35 was found^33^. CWD-A is regarded as probably efficacious because studies were conducted by only one research group, solely within the American population and not with clinically referred adolescents^34^. Research on clinically referred adolescents is necessary to enhance generalizability to clinical practice.

In addition, knowledge about possible moderators of treatment is desperately needed, but scarce^16,34^. A systematic review of randomized or controlled depression studies concluded that predictors and moderators were investigated in only a few studies (*n* = 13) since 2000, and outcomes provided little consistent knowledge about moderators^21^. It was concluded that gender, age, comorbidity, externalizing problems did not predict or moderate treatment results in depressed adolescents. Severity of depression and comorbid anxiety seem to be potential predictors of worse treatment outcome^21^. However, comorbide diagnoses are often used as exclusion criteria in efficacy trials and therefore not studied thoroughly.

Another literature review on CBT treatment studies on depressive youth found that treatment outcome was predicted by age, general functioning, and number of diagnoses and moderated by family income and severity of depression^35^. Inconsistency in findings may result from moderator relationships that are specific for one population, one outcome, one treatment or one specific moderator, but do not hold automatically in other circumstances^36^. Despite this restriction, the study of potential moderators, such as age, gender, educational level of the child and the parent, suicidal criteria, as well as comorbidity and severity of depression, is relevant within routine mental health care, because it can reveal essential knowledge for the usefulness of CBT in specific populations.

In conclusion, the efficacy of CBT as treatment of clinically depressed adolescents is well established, but the effectiveness in routine mental health care in comparison to other psychotherapies is not yet clear. Also, potentially important moderators have not been studied in effectiveness trials.

**Figure 1:**
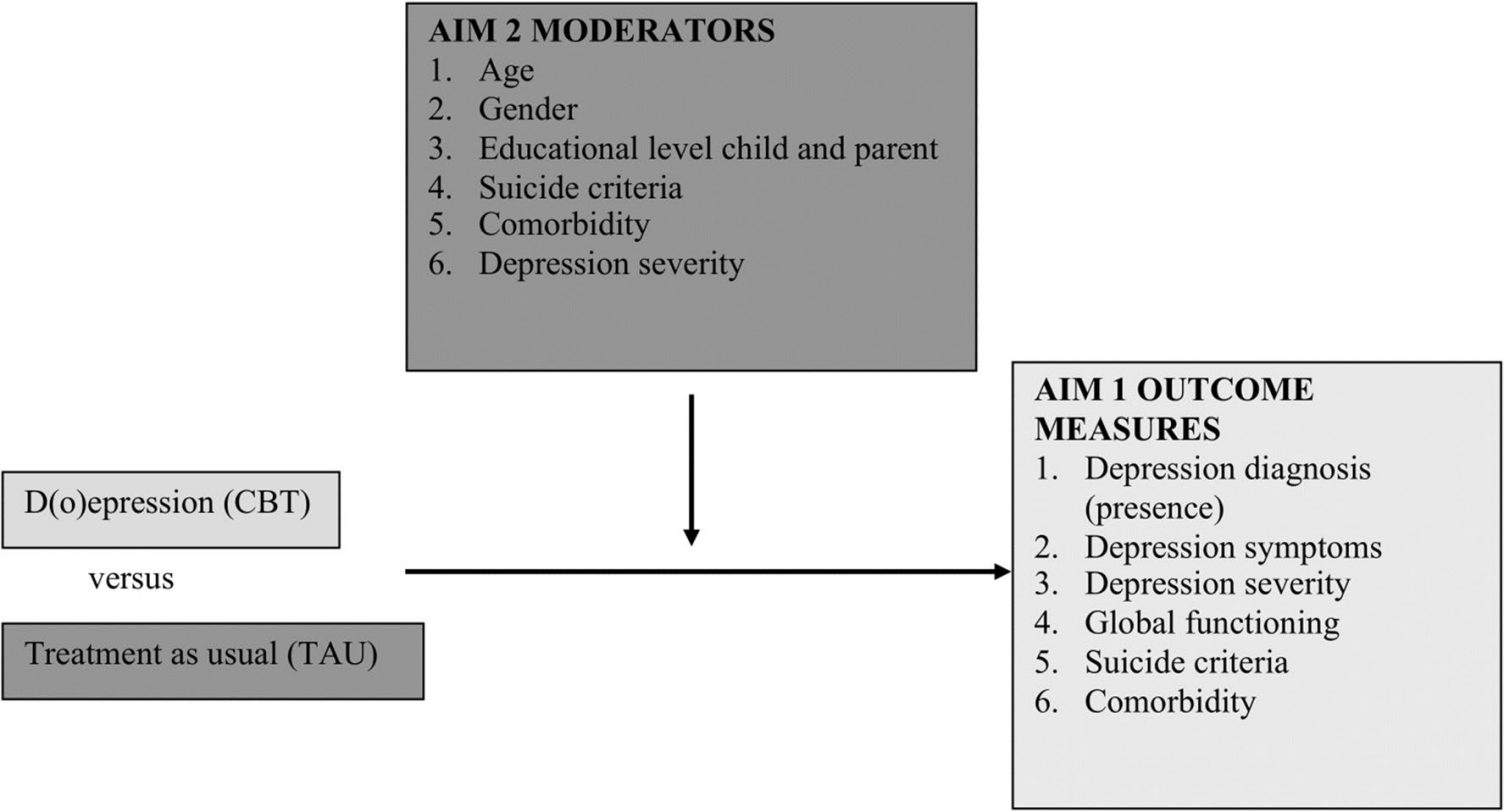
Aims and potential moderators evaluated in this study.

In this study, a randomized controlled trial was conducted investigating the effectiveness of the individual CBT program the “D(o)epression course” compared to TAU in a sample of clinically referred adolescents with a Major Depressive Disorder or Dysthymic disorder according to DSM-IV-TR^37^. The aims of this study were to investigate the (1) effectiveness and (2) potential moderators and predictors (age, gender, educational level of the adolescent and the parent, suicide criteria, comorbidity and severity of depression) of CBT versus TAU (see Fig. 1). CBT was expected to be more effective than TAU (without CBT). No specific hypotheses were formulated concerning the moderators due to inconclusive findings on moderators in previous research^21,38^.

## Method

### Study design

This study was designed as a multi-site (*N* = 14), randomized controlled clinical trial in which individual CBT was compared to Treatment as usual (TAU). Randomization was conducted after pre-treatment assessment and was executed per adolescent by computer generated block randomization and stratified per mental health care center by the primary researcher who did not conduct the interviews or any measurement. The study design is described in detail elsewhere^39^. The study design was approved by the independent Medical Ethical Committee of Utrecht Medical Centre at Utrecht University, number 10/446 and all research was performed in accordance with relevant guidelines and regulations. The trial was preregistered at the Dutch Trial register (NTRnumber 2676 (03/01/2011); www.trialregister.nl).

The following assessments took place; prior to treatment (pretreatment assessment), within treatment (mediator assessments), immediately after treatment or after 15 sessions (post treatment assessment), six months after treatment (six-month follow-up) and 1 year after treatment (1 year follow-up). In this paper, we will present the post treatment and six-month follow-up results. Assessments were multi-method (semi-structured interviews, questionnaires and ratings) and involved multiple informants (adolescent, parent and therapist). Independent research assistants who were blind to condition conducted semi-structured interviews. Questionnaires were completed online using Survalyzer (an online questionnaire server) at home by the adolescent and the parents using a separate login code to secure privacy. The therapist completed the questionnaires at the office.

The primary outcome measure was presence of a depression diagnosis based on a semi-structured diagnostic interview, the Kiddie-Schedule for Affective Disorders and Schizophrenia, present and lifetime version^40^. Secondary outcomes included depressive symptoms, severity of depression, global functioning, suicide risk, and comorbidity.

Based on previous research an effect size (Cohen’s *d*) of 0.53 was expected^18^. Power calculations in STATA indicated that 70 adolescents per condition (assuming an alpha of 0.05, a statistical power, 1-beta, of 0.80 and a drop-out of 20% would be required to detect a difference in depression diagnosis between conditions.

### Sample characteristics

**Figure 2:**
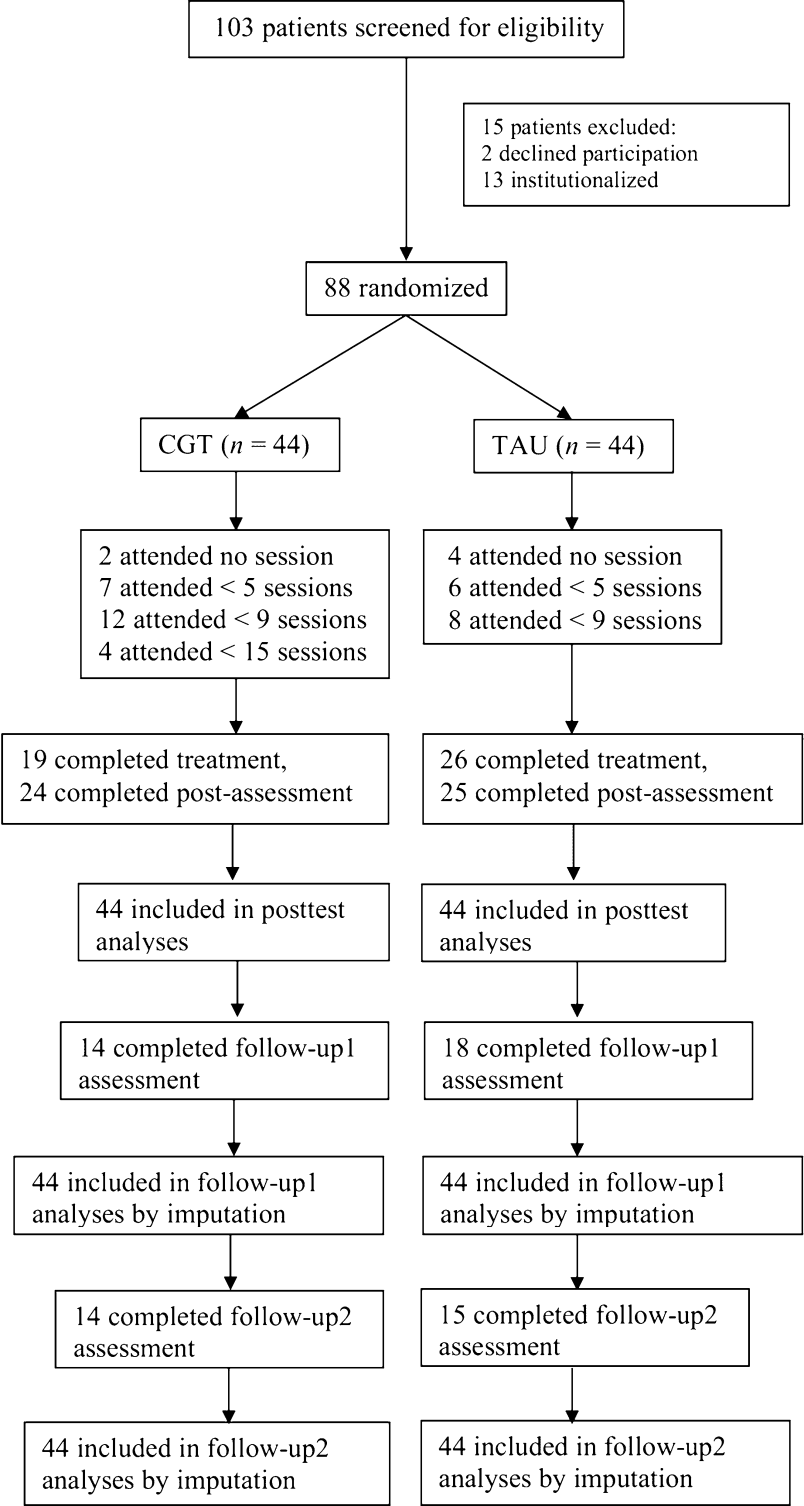
Participants flow chart.

In total, 103 referred clinically depressed adolescents, aged 12 to 21 and their parents (*n* = 71) were included in this study between 2011 and 2014 as planned. For a presentation of patient flow through the trial, see Fig. 2.

The participants were recruited from 14 different public mental health clinics geographically spread out over the Netherlands, from December 2011 to December 2014.

A total of 88 participants were randomized, their characteristics are shown in Table 1 and based on pre-treatment data. The mean age of the participants was 16.6 years (*SD* = 2.1) and 71% was younger than 18 years. Most participants (92%) were of Dutch origin and 2.3% of other European countries, 1.4% of Iran, 1.4% African and 2.9% unknown. The majority (82%) of the adolescents was female.

**Table 1:** Sample characteristics at pre-treatment and pre-treatment comparison between CBT and TAU.

Characteristics at pre-treatment	*n*	CBT	*n*	TAU	Test statistic
Female, *n* (%)	44	34 (77.3)	44	38 (86.4)	χ^2^ = 1.22
Age, mean (*SD*)	44	16.9 (2.2)	44	16.3 (2.0)	*t* = − 1.27
Dutch nationality, *n* (%)	41	39 (95.1)	43	42 (97.7)	χ^2^ = 0.40
Education level parents, *n* (%)	35		32		χ^2^ = 16.73**
Primary education only		1 (2.9)		0 (0.0)	
Secondary education: Low level		0 (0.0)		7 (21.9)	
Middle level		14 (40.0)		9 (28.1)	
High level		18 (51.4)		10 (31.3)	
University		2 (5.7)		6 (18.7)	
Education level children, *n* (%)	37		40		χ^2^ = 4.22
Secondary education: Special education		2 (5.4)		1 (2.5)	
Middle level		16 (43.2)		14 (35.0)	
High level		17 (46.0)		24 (60.0)	
University		2 (5.4)		1 (2.5)	
History of mental health service use, mean (*SD)*	35	2.8 (0.7)	40	2.7 (0.6)	*t* = − 1.20
Depression (CDI-II child version) mean (*SD*)	34	27.1 (8.7)	36	24.1 (6.7)	*t* = − 1.62
Depression (CDI-II parent version), mean (*SD*)	28	26.9 (8.0)	29	24.8 (6.4)	*t* = − 1.11
Depression severity (CGI-S) mean (*SD*)	37	4.16 (0.9)	37	3.92 (1.3)	*t* = − 0.95
Global functioning (CGAS), mean (*SD)*	37	49.5 (9.7)	35	49.4 (8.1)	*t* = − 0.08
Suicide criteria (SCA), mean (*SD*)	37	4.5 (3.9)	40	3.6 (3.2)	*t* = − 1.19
Number comorbid diagnoses, mean (*SD*)	41	0.83 (1.)	44	1.25 (1.8)	*t* = − 1.28

CDI-II = Child Depression Inventory-II; CGI-S = Clinical Global Impression-severity scale; CGAS = Children Global Assessment Scale; SCA = Suicide Criteria Assessment; CBT = Cognitive-Behavioral Therapy; TAU = Treatment as Usual.

*p ≤ .05, **p ≤ .01.

If the adolescent was 18 years or older, parents were only approached after the adolescent’s permission. Participation of parents was not always possible because some adolescents (*n* = 14) above the age of 18 did not consent to the involvement of parents in the treatment or in the study. If both parents participated, we included the parent who filled out most questionnaires during the study and had the most complete assessments. In total 71 parents were included, of which 10 had a child aged 18 or above. All participating parents were biological parents, their mean age was 48.4 (*SD* = 5.3) and 18.6% (*n* = 11) was male.

An experienced psychologist within the mental health center assessed the CDI-2 child and parent version to screen for depressive symptoms and informed the adolescents and their parents about the study. After written informed consent was given by the adolescent and his/her parents, a trained independent research assistant carried out the semi-structured diagnostic interview (KSADS) and checked whether the adolescent met the inclusion or exclusion criteria. The inclusion criteria were: (1) a primary diagnosis of Major Depressive Disorder or Dysthymic disorder, (2) age 12 to 21 years, and (3) referred to one of the participating mental health care institutions. The exclusion criteria were (1) acute suicide risk (2) substance abuse, pervasive developmental disorder, or bipolar disorder which required a different treatment approach (3) day care or admission to an inpatient clinical setting and (6) not fluent in Dutch, Turkish, Arabic or Berber language. If medication (for the depression or another disorder) was used in the CBT condition, the dosage at pre-treatment was kept constant during the intervention.

### Interventions

*CBT.* The experimental treatment consisted of the D(o)epression course^24^, which is an individual CBT protocol based on the CWD-A^23^. This treatment was limited to 15 weekly sessions of 45 min with the adolescent.

In line with the CWD-A, the D(o)epression course is based on the social learning theory, which explains the etiology of depression, as was developed by Lewinsohn and colleagues^22^. This theory states that there is a connection between the number of positive interactions between a person and his environment on one hand, and depression on the other. A triggering event such as a stressful life event may cause a negative spiral of less positive interactions, which in turn leads to more negative thoughts and a deteriorating depressed mood. The intervention aims to reduce depressive complaints in adolescents with a depressive disorder. The focus of the intervention is broad, since depressive episodes are considered to be multi-factorially determined. The CBT treatment is described in detail per session (what, how, when and how long) in a manual for the therapist. A workbook for the participant with psychoeducation, exercises and worksheets was also provided. The intervention contains representative CBT components^41,42^ namely: psycho-education (information about depression and the rationale for the etiology of the complaints and the treatment of them), setting attainable goals (translate large goals into realistic short term goals), self-monitoring (registration of the mood, activities and thoughts), activation (planning frequent, joyful activities), improving social skills and communication skills (improvement and stimulation of social behavior), relaxation techniques, cognitive restructuring (identifying and changing unrealistic negative thoughts about the self, others and events), role play and problem solution skills (teaching the creation of solutions for problems via brainstorm, choosing, trying and evaluating) and relapse prevention. The parents received psycho education and information on CBT after session three and nine. By the end of treatment a meeting with the parent(s) was scheduled to evaluate the treatment.

Treatment integrity was enhanced, by a standardized training in delivering the D(o)epression course and routine peer and expert supervision within the mental health care center.

TAU, the control treatment, for clinical depression was delivered by registered and qualified therapists specialized in different therapies. TAU consisted of 15 weekly sessions of 45 min until T1. A broad range of different treatments and combinations were delivered within the control condition: IPT, family therapy, medication, psychodynamic therapy (short duration), (non-directive) counseling and Eye Movement Desesititation and Reprocessing (EMDR) for depression , solution focused. See results under Treatment received. The TAU therapists received the same information about depressive disorders as the CBT therapists and were instructed not to use any CBT elements within the TAU condition. TAU treatment could continue after 15 sessions.

To enhance external validity, all sessions took take place within routine outpatient care in public mental health care institutions.

*Treatment integrity* was established by rating video and audio recordings of two randomly chosen sessions per treatment. Two trained psychologists performed these ratings. A treatment integrity scale was developed containing three factors, namely quality of the therapist (e.g. empathy, motivating client, instigating interaction, avoiding depressive talk), content of treatment (e.g. following protocol, attaining goals, instigate exercise like role play, modeling, providing feedback) and structure of the session (e.g. setting agenda, following time schedule, efficient use treatment time). Items were rated on a three-point scale (0 = absent, 1 = minimal, 2 = largely, 3 = maximal). Interrater reliability was established by rating the same sessions *(n* = 8) by the observers and a supervisor specialized in this protocol. The Intraclass Correlation Coefficient (ICC) was 0.92, which indicates excellent interrater reliability. The mean score on the treatment integrity was 2.68 (*SD* = 0.75) indicating that treatment integrity was good.

### Therapists

Treatments in both conditions were delivered by psychologists with at least one year of experience within professional mental health care. The therapist in the CBT condition had also at least one year of experience in conducting CBT. Therapists were not allowed to conduct treatments in both conditions.

In the CBT condition 37 therapists participated, and in the TAU condition 37 other therapists participated. Their mean age was 42.78 (*SD* = 11.47), 68.2% was female, most (98.6%) were originally Dutch, white Caucasian, and had on average 16.3 (*SD* = 7.16) years of experience. No differences were found for these therapist characteristics between both conditions.

The therapists within the CBT condition were in general CBT oriented (*n* = 27, 72.97%), eclectic (*n* = 9, 24.3%) or cognitive oriented (*n* = 1, 2.7%). Of these therapists, nine (23.4%) were registered as a CBT therapist. The therapists in the CBT condition received a two day training in delivering the D(o)epression course by the first author.

The overall orientation of TAU therapists was eclectic (*n* = 12, 32.4%), cognitive (*n* = 12, 32.4%), psychodynamic (*n* = 9, 24.3%), family based (*n* = 2, 5.4%) or another orientation (*n* = 2, 5.4%). Therapists in the TAU condition did not have CBT as a primary orientation of treatment.

### Instruments

The *primary outcome measure* was presence of a major depression or dysthymic diagnosis, assessed by a semi-structured diagnostic interview, the Kiddie-Schedule for Affective Disorders and Schizophrenia, present and lifetime version (K-SADS)^43,44^. The K-SADS assesses a wide range of diagnoses (present and life time). The view of the adolescent, the parent and the independent clinician were taken into account in the final diagnosis, expressed as diagnosis present (yes) or absent (no). In previous research, the concurrent and convergent validity of the K-SADS was supported^40,43^, the interrater agreement was high (range: 93% to 100%) and test –retest reliability alpha coefficients were excellent (0.77 to 1.00)^43^.

As *secondary outcome measures* depressive symptoms, severity and global functioning, suicide risk, and comorbidity were used.

The *degree of depressive symptoms* was measured by means of self-report using the Child Depression Inventory-II (CDI-2)^45,46^. The CDI-2 is a revision of the CDI^47,48^, which was translated in Dutch^45^. The CDI-2 child version consists of 28 items, which assess cognitive, affective and behavioral symptoms of depression in the past two weeks. Each item has three graded alternatives of which one is chosen (e.g., “0 = I feel like crying once in a while, 1 = I feel like crying many days, 2 = I feel like crying every day”). Higher scores reflect more depressive symptoms. The reliability of the CDI-2 total score was good in the current study (*α* = 0.82).

The CDI-2 also provides a parent version^46^, which contains 17 items. Parents have to indicate how often the adolescent has experienced cognitive, affective and behavioral symptoms in the past two weeks on a Likert scale ranging from 0 (not at all) to 4 (most of the time). The reliability of the total scale in de current study was good (*α* = 0.78).

S*everity of depression* was rated by the therapist on the one item Clinical Global Impression-severity scale (CGI-S)^49^ with a scale range from 1 (no complaints) to 7 (most severe depressive complaints).

*Global functioning* of the adolescent was assessed by the therapist on the Children Global Assessment Scale (CGAS)^50,51^. The therapist rated the overall quality of functioning of the adolescent in several contexts namely at home, at school, with friends and during leisure time. The scale contains one item and the scores range from 0 (extremely impaired functioning) to 100 (very good functioning).

*Suicide criteria* were assessed with the Suicide Criteria Assessment (SCA)^52^. The scale was constructed based on the Suicidal Ideation Questionnaire^53^ and the Columbia-Suicide Severity Rating scale^54^. The scale contains five questions including “I thought about killing myself” or “I thought about planning to kill myself”. These questions tap into different aspects of suicidal criteria such as the frequency of thoughts, wishes, plan and urges to perform action within the past two weeks on a scale from 0 (not at all) to 2 (nearly every day). The reliability of the SCA is good (*α* = 0.89).

*Comorbid psychopathology* was assessed on two levels namely on diagnosis level with the K-SADS (present or absent) and on symptom level with the Youth Self Report scale (YSR)^55^ for adolescents and the Child Behavior Check List (CBCL) for parents^55,56^. The YSR and CBCL use a three point Likert scale (0 = not true, 1 = somewhat or sometimes true, and 2 = very true or often true). A higher score indicates more symptoms of psychopathology. The YSR and the CBCL allow a distinction between internalizing and/or externalizing psychopathology. Reliabilities for the YSR internalizing problems (*α* = 0.79) and externalizing problems (*α* = 0.86) were good, as were the reliabilities for the CBCL scale for internalizing (*α* = 0.87) and externalizing (*α* = 0.85) problems.

*Demographic information* was gathered by questions about age, gender, ethnicity, education level and family income.

### Statistical analysis

All missing values were imputed at item and assessment level, except for the semi-structured interview. Imputations were generated in R version 3.2.1^57^ with package MICE version 2.22^58^. The imputation routine was tailor-made to the analysis problem and the predictor matrix and imputation method are optimized for efficiency. Initially, for each incomplete variable, the 10 best predictors were selected. Where needed, this selection has been extended to make the imputation model compatible to the analysis model. The experimental condition was always included as a predictor. Ten imputed datasets were generated with 25 iterations for the algorithm to converge.

Predictive mean matching^59^ was used as the imputation routine for continuous and categorical data. Predictive mean matching draws imputation from the observed data and is known to preserve patterns and relations that are present in the data^60^. Variables that contained bonafide missings, such as items that are not applicable for a particular respondent, were imputed with a custom adaptation of predictive mean matching. The analyses were pooled based on Rubin’s rules^61^ following the work from^62^. Intent-to-treat as well as completer analyses were conducted on the secondary measures.

Pre-treatment differences were analyzed with chi-square tests for dichotomous variables and *t*-test for continuous variables. Within group effects were examined by looking at the percentage of adolescents that did not meet the required criteria of the depressive or dysthymic disorder as indicated by the K-SADS. Continuous variables were analyzed using paired *t*-tests. To calculate the effect sizes within the conditions, the formula described in Dunlap, Cortina, Vaslow, and Burke^63^ was used. The criteria used to interpret the effect sizes (g) were: 0.10 = small, 0.30 = small-to-moderate and 0.50 = a large effect^64^. Clinical significance was established by examining the percentage of adolescents falling below the clinical cut-off score of 14 for the CDI-2 for the child version^45,46^, and 17 for the parent version^46^.

Between group effects were analyzed with chi-square tests on the percentages of adolescents that did not meet all criteria of the depressive or dysthymic as indicated by the K-SADS. Between group effects for continuous variables at post treatment and 6 month follow-up were evaluated, using ANCOVAs with pre-treatment scores as a covariate to control for pre-treatment differences, as recommended^65,66^. TAU versus CBT effect sizes were computed as described by Hedges and Olin^67^ using a statistical software package named Comprehensive Meta-analysis, version 3.3.070.

Moderation was analyzed by multi-group analyses for dichotomous variables and linear regression analyzes for adolescent or parent reported continuous variables. In view of the number of participants in relation to the number of variables, separate analyses had to be used. Moderation was studied by separate linear regression analyses for condition on adolescent or parent reported depressive symptoms at post treatment and at 6 month follow-up while controlling for pre-treatment depressive symptoms and adding a potential predictor and the interaction variable. The tested potential predictors and moderators were adolescent age, adolescent gender, adolescent and parent education level, suicidal criteria, adolescent and parent reported comorbid externalizing problems, and therapist rated depression severity.

## Results

### Sample

The level of depressive symptoms reported by the adolescent (*N* = 70) on the CDI-2 at pre-treatment was high (*M* = 25.6; *SD* = 7.8), considering the cut-off score of 14, therefore the participants scored high above the level of clinical significance (Kovacs, 2011). More than half of the participants had at least one-comorbid diagnosis (58%, *n* = 49) on the semi-structured interview (*N* = 85). Most frequent disorders were Social phobia (*n* = 21, 25%), Generalized Anxiety Disorder (*n* = 23, 27%), Specific Phobia (*n* = 7,8%), Panic Disorder (*n* = 5, 6%), Separation Anxiety Disorder (*n* = 4, 5%), Post Traumatic Stress Disorder (*n* = 6, 7%), Attention Deficit Hyperactivity Disorder (*n* = 11, 13%) and Oppositional Defiant Disorder (*n* = 3, 4%). On the K-SADS, 72% (n = 61) reported thinking about death and 58% (n = 49) about suicidal ideation. Half of the sample had previous experience with mental health services (53%, n = 45).

Pre-treatment differences in the non-imputed data are reported in Table 1. Groups did not differ significantly on gender, age, ethnicity, history of mental health care use, internalizing- or externalizing problems (YSR/CBCL), depressive symptoms (CDI-2), pre-treatment number of diagnoses (K-SADS), suicide criteria assessment (SCA), global functioning (CGAS) or severity of depression (CGI-S). The chi-square test showed that only parental level of education in the CBT condition was significantly higher than in the TAU condition. Overall, randomization can be considered as succeeded.

### Treatment received

The number of treatment sessions in both conditions between pre- and post treatment assessment did not differ (*M*_TAU_ = 12.8, *SD* = 5.5; *M*_CBT_ = 12.7, *SD* = 4.8). At pre-treatment, 9% of the adolescents in the CBT condition and 13.6% in the TAU condition already received antidepressants, a nonsignificant difference. See the result section treatment received. The content of Treatment as Usual is provided were eclectic 40.9% (*n* = 18), IPT 25% (*n* = 11), Eye Movement Desensitization and Reprocessing 11.4% (*n* = 5), Solution focused therapy 2.3% (*n* = 1), Family therapy 2.3% (*n* = 1), group counseling 2.3% (*n* = 1), medication only 2.3% (*n* = 1) and some did not start treatment 13.6% (*n* = 6). Within the TAU condition 11% (n = 5) changed to treatment with medication only during treatment.

### Drop-out and adverse events

In CBT, more adolescents (57%, *n* = 25) discontinued treatment compared to TAU (41%, *n* = 18), but this difference was not significant (χ^2^(2) = 2.236, *p* = 0.327). Of these drop-outs, 22 (50%) in the CBT group and 16 (36%) in the TAU group dropped out during treatment. Different reasons led to discontinuation of treatment. In TAU, the reasons for discontinued treatment were elevation of depressive symptoms (38.8%, *n* = 7), lack of motivation (33.8%, *n* = 6), and reason unknown (27.7%, *n* = 5). In the CBT condition, discontinuation of treatment occurred because of change to another psychological intervention (28%, *n* = 7), reason unknown (24%, *n* = 6), elevation of depressive symptoms (20%, *n* = 5), change to medication (16%, *n* = 4) and lack of motivation (12%, *n* = 3).

Adolescents who discontinued CBT or TAU did not differ in gender or education level. However, an independent-samples t-test showed a significantly higher level of pre-treatment depressive symptoms on the CDI-2 in the CBT participants who discontinued treatment (*M*_CBT_ = 29.3, *SD* = 9.0) compared to participants that discontinued TAU (*M*_TAU_ = 22.9, *SD* = 6.9), *t*(29) = -2.2, *p* = 0.04, partial eta squared = 0.4).

Three adverse events occurred in the CBT condition (and none in the TAU condition), that is two suicide attempts (4.5%) before session 5, and one participant committed suicide (2.2%) between 6 month follow-up and 1 year follow-up. In the latter case, the therapist and parents had observed good response to CBT and a maintenance dosage of medication was given as recommended by the Dutch clinical guidelines^68^.

### Treatment effectiveness

#### Primary outcome

The clinical diagnostic interviews (KSADS) were conducted with 54 adolescents (61%) at post treatment, and with 39 (38%) at follow-up. Within the CBT condition, 76% (*n* = 19) of the treatment-completing adolescents did not meet criteria for depressive or dysthymic disorder on the K-SADS at post treatment and 88% (*n* = 18) at 6 month follow up. Within the TAU condition, these percentages within completers were 76% (*n* = 22) and 79% (*n* = 15) respectively.

#### Secondary outcome

Paired-samples *t*-tests, two sided, were conducted to measure change within the CBT and TAU condition on secondary outcome measures reported by adolescent, parent or therapist and between pre- and post and pre and 6 month follow-up treatment. Effect sizes are reported in Table 2.

**Table 2:** Within and between condition pooled pre-posttreatment and pre-follow-up1 treatment effect sizes (*g*) for Adolescent (*n* = 88 ~) and Parent (*n* = 88 ~) Questionnaires.

Scale	Pre-post			Pre-6-month follow-up		
	CBT (*g)*	TAU (*g)*	CBT-TAU(*g*)	CBT (*g*)	TAU (*g*)	CBT-TAU (*g*)
CDI-II Child	0.92***	0.69***	0.20	1.37***	1.37***	0.09
CDI-II Parent	0.47**	0.71**	− 0.18	0.91***	1.15***	− 0.15
CGI-S^a^	0.77**	1.14***	− 0.26	––	–	–
CGAS^a^	0.58**	1.07***	− 0.27	–	–	–
SCA	0.48**	0.26	0.08	0.05***	1.13***	− 0.40
YSR INT	0.70***	0.83***	0.01	1.31***	1.22***	0.15
YSR EXT	0.19	0.34	− 0.25	0.96**	0.60**	− 0.17
CBCL INT	0.10	0.55**	− 0.55	0.67***	1.00***	− 0.32
CBCL EXT	0.05	0.11	− 0.29	0.09	0.44*	− 0.29

CDI-2 = Child Depression Inventory II; Child = child version; Parent = Parent version; CGI-S = Clinical Global Impression-severity scale; CGAS = Children Global Assessment Scale; SCA = Suicide Criteria Assessment; YSR = Youth Self Report scale; INT = internalizing problems; EXT = externalizing problems; CBCL = Child Behavior Check List; CBT = Cognitive-Behavioral Therapy; TAU = Treatment As Usual. ~ Data were multiple imputed.

^a^No 6-month follow-up measurements of the CGAS and CGI-S were collected.

*p < .05, **p < .01, ***p < .001.

Intent to treat analyses on imputed data showed a statistically significant decrease on depressive symptoms (CDI-2) reported by the adolescent within both conditions, respectively within the CBT condition pre-post (*t*(96) = 5.9, *p* < 0.001) and pre- to 6 month follow-up (*t*(54) = 9.2, *p* < 0.001) and the TAU condition pre-post (*t*(39) = 4.06, *p* < 0.001) and pre-6 month follow-up (*t*(34) = 8.14, *p* < 0.001). The effect sizes varied from moderate to large, see Table 2. Also, a clinically significant reduction in depressive symptoms (below the cut off score on the CDI-2) at post treatment was reached in 41.6% (*n* = 18) of the cases within the CBT condition and 31.8% (*n* = 14) within the TAU condition. At 6 month follow-up, 61.4% (*n* = 27) within the CBT and 47.7% (*n* = 21) within the TAU condition had a CDI-2 score below the clinical cut-off score.

Intent to treat analysis showed a significant decrease in parent report of adolescent depressive symptoms within the CBT condition from pre to post (*t*(21) = 3.48, *p* = 0.002) and pre to 6 month follow-up treatment (*t*(15) = 5.99, *p* < 0.001), with a moderate and large effect size respectively. A significant decrease was also found within the TAU condition, pre-post (*t*(18) = 3.81, *p* = 0.001) and pre-6 month follow-up (*t*(22) = 7.22, *p* < 0.001), with large effect sizes. Parents reported a clinically significant reduction in depressive symptoms in their child at post treatment in 35.9% (*n* = 16) within the CBT condition and 39.8% (*n* = 18) within the TAU. At follow-up, 52.5% (*n* = 23) within CBT and 61.8% (*n* = 27) within TAU condition had a CDI-2 score below the clinical cut-off score.

Ratings from the therapist showed that depression severity decreased while global functioning of the adolescent increased from pre to post treatment, with large effect sizes in both conditions (see Table 2).

Comorbid suicide criteria reported by the adolescent within CBT (*t*(132) = 2.97, *p* = 0.004) dropped significantly at post treatment but not in the TAU condition. At 6 month follow-up within both conditions a decrease in suicide criteria was found, respectively CBT (*t*(48) = 4.55, *p* < 0.001) and TAU (*t*(243) = 5.79, *p* < 0.001).

Internalizing symptoms (YSR) reported by the adolescent were also significantly reduced within both treatments, respectively within the CBT condition pre-post (*t*(62) = 4.56, *p* < 0.001) and pre- to 6 month follow-up (*t*(44) = 7.66, *p* < 0.001) and TAU condition pre-post (*t*(61) = 4.53, *p* < 0.001) and pre-6 month follow-up (*t* (46) = 5.96, *p* < 0.001).

Comorbid externalizing problems reported by the adolescent decreased significantly from pre- to 6 month follow-up, within CBT (*t*(38) = 2.98, *p* = 0.005) as well as in TAU(*t*(130) = 3.34, *p* = 0.001). Only parents in the TAU condition reported a significant decrease of externalizing symptoms from pre to follow-up1 (*t*(13) = 2.46, *p* = 0.021), while parents in the CBT condition reported no significant change.

### Effectiveness of CBT versus TAU

#### Primary outcome

Treatment completer analyses (*N*_TAU_ = 29_,_ *N*_CBT_ = 25) were performed. Chi-square tests were done to measure differences in the percentage of adolescents who did not meet all the criteria for depressive or dysthymic disorder. No significant difference was found between TAU and CBT at post treatment, χ^2^(1) = 0.000, *p* = 0.99 nor at follow-up1, χ^2^(1) = 0.914, *p* = 0.339.

#### Secondary outcomes

Between groups comparisons were conducted, using ANCOVAs with pre-treatment scores as a covariate, see Table 2. No main effect of condition was found on self-reported and parent reported depressive symptoms at post treatment, nor at 6 month follow-up. The effect sizes for pre-post and pre-6 month follow-up scores between the conditions were respectively small and very small, see Table 2. Also, no significant main effects of condition were found at post treatment or 6 month follow-up on the following variables; therapist rated depression severity and global functioning, adolescent reported suicide criteria and adolescent and parent reported internalizing and externalizing symptoms. Effect sizes varied between almost zero for adolescent reported internalizing problems at post treatment to moderate (0.55) for parent reported internalizing problems at post treatment between the two conditions.

### Predictors and moderators

Potential predictors and moderators were analyzed with linear regression analyses, with adolescent and parent reported depressive symptoms as the dependent variables. The following variables were included: adolescent age, adolescent gender, adolescent and parent education level, suicidal risk, adolescent and parent reported comorbid externalizing problems, and therapist rated depression severity. No significant predictors or moderators were identified.

## Discussion

In this study, CBT was compared to an active treatment condition (TAU), under rigorous conditions within routine care institutions and with a referred clinical sample of adolescents diagnosed with depression or dysthymia. Results can be summarized as follows. Both treatments showed a significant decrease in the percentage of adolescents who met the criteria for a depressive or dysthymic disorder as well as a significant decrease in self-reported and parent reported depressive symptoms with large effect sizes. No significant differences were found between the two conditions in depression diagnosis or on adolescent and parent reported depressive symptom level, both at post treatment or at six month follow-up. Also, no significant differences were found between CBT and TAU on therapist reported depression severity and global functioning, suicide criteria, and adolescent reported internalizing and externalizing comorbid symptoms. Although not significant, more adverse events occurred in CBT compared to TAU. No predictors or moderators of treatment were found regarding age, gender, educational level adolescent and parent, comorbidity and severity of depression.

The finding that treatment in both conditions showed a decrease in number of depression diagnoses and a clinically significant symptom reduction in this specific sample is reassuring.

The outcome of CBT treatment in a large RCT, the Treatment Adolescent Depression Study (TADS), conducted with a community recruited sample (*n* = 327) was found to be worse when severity of depression and comorbidity were high^38^. This could implicate that the outcome of CBT in a more severe and complex sample like ours, could be small. However, we did find a large effect size (*ES* = 0.92) at post treatment for CBT.

We hypothesized that CBT would outperform TAU, but it did not. Previous research showed that the group manual of CWD-A outperformed TAU in several RCT’s (*n* = 3)^33^. However, the finding of this study is in line with the results of a more recent meta-analysis showing that evidence-based protocols do not outperform usual care in clinically referred samples or in youths with a diagnosis, including depression^70^.

Several explanations for these contradictory findings can be provided. *First,* in our study, the control condition was TAU. In general, the quality of TAU is considered an important influential factor on the results of a RCT and specifically for effectiveness of CBT^71^. Moreover, structural aspects (such as number and duration of sessions, training of therapist, format of therapy and restriction of topics) have been shown to be important for the proportion of established effectiveness in placebo studies^72^. In our study, the quality of TAU was high. The therapists who performed TAU treatments in this study were highly qualified and were experienced in tailoring the treatment to the individual’s needs and discuss more broad topics than was allowed in CBT. Furthermore, the TAU consisted of a substantial amount of evidence-based treatments for depression, for instance IPT and anti-depressant medication or a combination of both. It is known that CBT does not outperform these evidence-based treatments. For example, CBT did not outperform IPT^20^ and medication outperformed CBT as acute treatment^73^. Besides, TAU in routine mental health care is also considered to be a stronger standard to test manualized protocols than other control conditions^70^.

*Second,* the treatment dose could not be kept equal in both conditions after post treatment. Some TAU treatments continued after 15 sessions in contrast to the CBT treatment, which ended after 15 sessions. Continuation of CBT after 15 sessions was a reason to drop-out of the study. It is possible that TAU needed more sessions to reach the level of decrease in depressive symptoms between post treatment and follow-up after six months. Another possibility is that the amount of reduction in depressive symptoms could partially be explained by the natural course of depression in participants in both treatment conditions. Spontaneous reduction of depressive symptoms can occur even without treatment after eight months, the mean duration of a major depressive disorder^13^.

*Third*, the amount of comorbid diagnoses was substantial in both conditions and interfered with CBT treatment delivery. In this study, the CBT manual did not allow for adaptations to address comorbidity. In the TAU condition the therapists were free to treat comorbid diagnoses as well, next to treating the depression. The content of TAU treatments compared to CBT may target behavior problems more specifically, which might be related to irritable mood as a characteristic of depression and therefor important to the treatment of depression. For example, parents in the TAU condition reported a significant decrease of externalizing symptoms at post treatment, whereas parents in the CBT condition did not.

Furthermore, one-third of the adolescents discontinued CBT treatment and changed to another psychological treatment. TAU perhaps is better equipped to address comorbidity by adding other psychological treatment elements. Previous research found that the highly flexible CBT treatment containing different components used in the TADS study did not result in a better response rate namely 48% after 12 weeks and 65% after 18 weeks on the CGI-I^72^. However, discontinuation of CBT treatment in the TADS study was much lower than in the present study namely 15% after 12 weeks and 19% after 18 weeks. Probably a tradeoff between inflexibility and discontinuation is inevitable.

*Fourth*, another explanation is the adaptation of the group-based manual of CWD-A into an individual based manual without any peer interaction which could have influenced effectiveness. Group treatments are considered to work better than individual treatment in depressed teens^42^.

Fifth, last but not least, common factors (alliance, empathy, expectations and therapist differences) were active in both conditions. Evidence from a large review supports that common factors contribute to the benefits of a treatment^74^. Therefor the common factors should also be considered as a possible explanation for the outcome instead of the specific ingredients of CBT or TAU.

Since no differences in effectiveness were found, we still need to determine which treatment a therapist preferably should use when dealing with adolescent depression. There are several factors that may be considered for this decision.

Besides effectiveness, this study examined possible moderators of treatment, which could be indicative of selecting a specific treatment for a specific patient. Search for moderators is important in order to identify sources of individual differences in treatment response and guide differential treatment selection. However, no effect of moderators could be established and thereby no specific group whom benefited more from CBT could be identified. The sample size (low power) may be accountable for this result^38^. Apart from sample size another statistical reason should be considered. Kraemer^36^ pointed out that research on a heterogeneous population with major individual differences is more likely to find moderators with a sensitive measure for individual differences^21,36^. However, the group within this study was rather homogeneous with regard to demographics and diagnoses which decreases the chance of identifying a moderator. For instance, the education level of the participants was not significantly different between the experimental and control condition and variance within the condition was rather small.

Apart from moderators, cost-effectiveness of different treatments could also form a factor to take into account when choosing for a specific treatment. The cost effectiveness of CBT versus TAU will be presented in a forthcoming paper and was not further examined for this paper.

Also, speed of symptom reduction within both conditions is important for the selection of treatments. Depression in adolescents should be treated as soon as possible and aggressively in order to shorten the depressive period^68^. Reduction of the time to response has the potential to diminish the risk for suicide, functional impairment, substance abuse and problems at school and work as much as possible^69^. In future research, trajectories of treatment response should be analyzed.

Future research should also address mediators of outcome in CBT and TAU. For instance, reduction of negative thoughts by cognitive restructuring may mediate the relation between pre and post treatment depressive symptoms in the CBT condition.

A finding of serious concern was the high level of self-reported depressive symptoms at the time of referral. Of the adolescents 53% already received treatment before entering the mental health center. Although mental health treatment of children under 18 is free of charge in the Netherlands, a significant amount of depressed adolescents only sought treatment when depression was already severe. The dissemination and organization of effective treatment still needs attention to reach out to depressed adolescents in time.

This study also has several limitations. *First,* our sample size was not as big as was advised by our power calculation. We included 88 adolescents while the power calculation recommended 140 adolescents. Also, the effect size of TAU in mental health institutions in the Netherlands was found to be larger than TAU in studies conducted in other countries and settings on which the power calculations were based. As a result the power of the study was not as high as preferred.

*Second,* the amount of participants with a low education level or with a different ethnic background was small. Even though this is representative for the population in youth mental health care in the Netherlands, the generalization of the findings is limited to this particular group.

*Third*, even the adolescents and parents who decided to participate in the study were found to be hesitant to participate due to the extra assessments. Although assessments were conducted online and were easy to monitor, it was difficult to reach full response of all participants.

*Fourth*, TAU was not limited to 15 sessions. The number of sessions in TAU after post treatment (15 sessions) is not clear because the therapists sometimes did not register the forms completely. The total amount of face-to-face contact between post treatment and followup1 was probably larger within TAU than in CBT. This could have influenced the increased effect size in TAU.

*Fifth*, the participating institutions in this study may have differed in various ways, including in priority and affinity to conduct research. This may have affected the findings^70^.

*Sixth*, last but not least, common factors (alliance, empathy, expectations and therapist differences) were active in both conditions. Evidence from a large review, supports that common factors contribute to the benefits of a treatment^74^. Therefor the common factors should also be considered as a possible explanation for the outcomes instead of the specific ingredients of CBT or TAU. This is also important compared to studies using waitlist control groups, where common factors are not active in both conditions^74^.

## Conclusion

Treatment of depression or dysthymic disorders in adolescents between 12 and 21 years with CBT or TAU showed a significant reduction of depressive diagnoses as well as in the number of depressive symptoms. However, more than half of the completers in CBT and TAU condition still had an elevated level of depressive symptoms at post treatment. CBT did not outperform TAU in this complex and severe clinical sample. No moderators of treatment outcome were found.

Further studies are needed to establish cost-effectiveness of treatments and to identify mediators in order to improve treatment effectiveness by permitting personalizing treatment to address comorbidity in a systematic way.

## References

[1] World Health Organization. Global burden of mental disorders and the need for a comprehensive, coordinated response from health and social sectors at the country level. (Report by the Secretariat). Geneva: World Health Organization (2011).

[2] Kessler RC (2005). Lifetime prevalence and age-of-onset distributions of DSM-IV disorders in the national comorbidity survey replication. Arch. Gen. Psych..

[3] Thapar A, Collishaw S, Pine DS, Thapar AK (2012). Depression in adolescence. The Lancet..

[4] Cohen P (1993). An epidemiological study of disorders in late childhood and Adolescence—I. Age-and Gender-Specific prevalence. J. Child Psychol. Psychiat..

[5] Fergusson DM, Horwood LJ, Lynskey MT (1993). Prevalence and comorbidity of DSM-III-R diagnoses in a birth cohort of 15 year olds. J. Am. Acad. Child. Adol. Psychiat..

[6] Hankin BL (1998). Development of depression from preadolescence to young adulthood: emerging gender differences in a 10-year longitudinal study. J. Abnorm. Psychol..

[7] McGee R (1990). DSM-III disorders in a large sample of adolescents. J. Am. Acad. Child. Adol. Psychiat..

[8] Hoeymans, N., Gommer, A. & Poos, M. *Welke verschillen zijn er tussen leeftijdsgroepen: Volksgezondheid toekomstverkenning, nationaal kompas volksgezondheid.* (RIVM, 2006).

[9] Curry JF (2011). Recovery and recurrence following treatment for adolescent major depression. Arch. Gen. Psychiat..

[10] Ryan ND (2005). Treatment of depression in children and adolescents. The Lancet.

[11] Goodyer I, Cooper PJ, The clinical features of identified disorder (1993). A community study of depression in adolescent girls. II. Br. J. Psychiat..

[12] Portzky G, Van Heeringen C (2009). Suïcide bij jongeren. Psych Gez..

[13] Birmaher, B., Brent, D. & AACAP Work Group on Quality Issues. Practice parameter for the assessment and treatment of children and adolescents with depressive disorders*. J Am Acad Child Adol Psychiat.* **46**, 1503–1526 (2007). 10.1097/chi.0b013e318145ae1c 18049300

[14] McDermott, B. *et al*. *Clinical practice guidelines: Depression in adolescents and young adults.* (Beyondblue: the national depression initiative (2010).

[15] National Collaborating Centre for Mental Health (UK). *Depression in children and young people: Identification and management in primary, community and secondary care.* (The British Psychological Society & The Royal College of Psychiatrists, 2005). 21834190

[16] Weisz JR, McCarty CA, Valeri SM (2006). Effects of psychotherapy for depression in children and adolescents: a meta-analysis. Psychol Bull..

[17] Lewinsohn PM, Clarke GN (1999). Psychosocial treatments for adolescent depression. Clin. Psychol. Rev..

[18] Klein JB, Jacobs RH, Reinecke MA (2007). Cognitive-behavioral therapy for adolescent depression: a meta-analytic investigation of changes in effect-size estimates. J. Am. Acad. Child. Adol. Psychiat..

[19] Watanabe N, Hunot V, Omori I, Churchill R, Furukawa T (2007). Psychotherapy for depression among children and adolescents: a systematic review. Acta. Psychiatr. Scand..

[20] Zhou X (2015). Comparative efficacy and acceptability of psychotherapies for depression in children and adolescents: a systematic review and network meta-analysis. World Psychiat..

[21] Nilsen TS, Eisemann M, Kvernmo S (2013). Predictors and moderators of outcome in child and adolescent anxiety and depression: A systematic review of psychological treatment studies. Eur. Child. Adol. Psychiat..

[22] Lewinsohn PM, Antonuccio DO, Steinmetz J, Teri L (1984). The coping with depression course: a psychoeducational intervention for unipolar depression.

[23] Clarke GN, Lewinsohn PM, Hops H (1990). Adolescent coping with depression course.

[24] Stikkelbroek Y, Bouman H, Cuijpers P (2005). De doepressiecursus.

[25] Clarke GN, Rohde P, Lewinsohn PM, Hops H, Seeley JR (1999). Cognitive-behavioral treatment of adolescent depression: efficacy of acute group treatment and booster sessions. J. Am. Acad. Child Adol. Psychiat..

[26] Clarke GN (2002). Group cognitive-behavioral treatment for depressed adolescent offspring of depressed parents in a health maintenance organization. J. Am. Acad. Child. Adol. Psychiat..

[27] Clarke GN (2009). Randomized effectiveness trial of an internet, pure self-help, cognitive behavioral intervention for depressive symptoms in young adults. Cogn. Beh. Ther..

[28] Lewinsohn PM, Clarke GN, Hops H, Andrews J (1990). Cognitive-behavioral treatment for depressed adolescents. Beh. Ther..

[29] Rohde P, Clarke GN, Mace DE, Jorgensen JS, Seeley JR (2004). An efficacy/effectiveness study of cognitive-behavioral treatment for adolescents with comorbid major depression and conduct disorder. J. Am. Acad. Child. Adol. Psychiat..

[30] Rosselló J, Bernal G (1999). The efficacy of cognitive-behavioral and interpersonal treatments for depression in puerto rican adolescents. J. Cons. Clin. Psychol..

[31] Rosselló J, Bernal G, Rivera-Medina C (2008). Individual and group CBT and IPT for puerto rican adolescents with depressive symptoms. Cult. Divers. Ethn. Minor Psychol..

[32] Clarke GN (2005). A randomized effectiveness trial of brief cognitive-behavioral therapy for depressed adolescents receiving antidepressant medication. J. Am. Acad. Child Adol. Psychiat..

[33] Cuijpers P, Muñoz RF, Clarke GN, Lewinsohn PM (2009). Psychoeducational treatment and prevention of depression: the “Coping with depression” course thirty years later. Clin. Psychol. Rev..

[34] David-Ferdon C, Kaslow NJ (2008). Evidence-based psychosocial treatments for child and adolescent depression. J. Clin. Child. Adol. Psychol..

[35] Curry JF (2006). Predictors and moderators of acute outcome in the treatment for adolescents with depression study (TADS). J. Am. Acad. Child. Adol. Psychiat..

[36] Kraemer HC (2013). Discovering, comparing, and combining moderators of treatment on outcome after randomized clinical trials: a parametric approach. Stat. Med..

[37] American Psychiatric Association (2000). Diagnostic and statistical manual-text revision (DSM-IV-TR).

[38] Curry JF (2009). Research psychotherapy: aspirin or music?. Clin. Psychol. Sc. Pract..

[39] Stikkelbroek Y, Bodden DH, Dekovic M, van Baar AL (2013). Effectiveness and cost effectiveness of cognitive behavioral therapy (CBT) in clinically depressed adolescents: individual CBT versus treatment as usual (TAU). BMC Psychiat..

[40] Lauth B (2010). Validity of K-SADS-PL (schedule for affective disorders and schizophrenia for school-age children-present and lifetime version) depression diagnoses in an adolescent clinical population. Nord. J. Psychiat..

[41] McCarty CA, Weisz JR, Hamilton JD (2007). Effects of psychotherapy for depression in children and adolescents: what we can (and can't) learn from meta-analysis and component profiling. J. Am. Acad. Child Adol. Psychiat..

[42] Weersing VR, Rozenman M, Gonzalez A (2009). Core components of therapy in youth: Do we know what to disseminate?. Beh. Modific..

[43] Kaufman J (1997). Schedule for affective disorders and schizophrenia for school-age children-present and lifetime version (K-SADS-PL): Initial reliability and validity data. J. Am. Acad. Child. Adol. Psychiat..

[44] Reichart C, Wals M, Hillegers M (2000). Vertaling K-sads.

[45] Bodden DH, Braet C, Stikkelbroek Y (2016). CDI-2, vragenlijst voor depressie bij kinderen en jongeren.

[46] Kovacs M (2011). The child depression inventory 2 (CDI-2).

[47] Braet C, Timbremont B (2002). Children’s depression inventory [Dutch version].

[48] Kovacs M (1992). Children's depression inventory: manual.

[49] Guy W (1976). *ECDEU assessment manual for psychopharmacology* (US Department of Health, Education, and Welfare, Public Health Service, Alcohol, Drug Abuse, and Mental Health Administration.

[50] Bunte T, Schoemaker K, Matthys W (2010). Children’s global assessment scale (CGAS) dutch translation.

[51] Shaffer DA (1983). children's global assessment scale (CGAS). Arch. Gen. Psychiat..

[52] Stikkelbroek Y, Bodden DH (2016). Suicide assessment scale.

[53] Reynolds W (1987). Professional manual for the suicidal ideation questionnaire.

[54] Posner K (2011). The Columbia-Suicide severity rating scale: Initial validity and internal consistency findings from three multisite studies with adolescents and adults. Am. J. Psychiat..

[55] Achenbach TM (1991). Integrative guide for the 1991 CBCL/4-18, YSR, and TRF profiles.

[56] Verhulst FC, van der Ende J, Koot JM (1996). Handleiding voor de CBCL/4–18.

[57] R Core Team. R. *A language and environment for statistical computing*. Retrieved from www.R-project.org (2015).

[58] Buuren, S., Groothuis-Oudshoorn, K. Mice: Multivariate imputation by chained equations in R. J *Statist Softw.* **45**. purl.utwente.nl/publications/78938 (01–06–2015).

[59] Little RJ (1988). Missing-data adjustments in large surveys. J. Bus. Econ. Stat..

[60] Vink G, Frank LE, Pannekoek J, Buuren S (2014). Predictive mean matching imputation of semicontinuous variables. Stat. Neerland..

[61] Rubin DB (2004). Multiple imputation for nonresponse in surveys.

[62] Li K, Meng X, Raghunathan TE, Rubin DB (1991). Significance levels from repeated p-values with multiply-imputed data. Stat. Sin..

[63] Dunlap WP, Cortina JM, Vaslow JB, Burke MJ (1996). Meta-analysis of experiments with matched groups or repeated measures designs. Psychol. Meth..

[64] Cohen J (1988). Statistical power analysis for the behavioral sciences.

[65] Rausch JR, Maxwell SE, Kelley K (2003). Analytic methods for questions pertaining to a randomized pretest, posttest, follow-up design. J. Clin. Child. Adol. Psychol..

[66] Vickers AJ, Altman DG (2001). Statistics notes: Analysing controlled trials with baseline and follow up measurements. BMJ Clin. Res. Ed..

[67] Hedges LV, Olkin I (2014). Statistical method for meta-analysis.

[68] Buitelaar J (2009). Addendum jeugd bij de MDR depressie DEF.

[69] Kratochvil C (2006). Acute time to response in the treatment for adolescents with depression study (TADS). J. Am. Acad. Child Adol. Psychiat..

[70] Weisz JR (2013). Performance of evidence-based youth psychotherapies compared with usual clinical care: A multilevel meta-analysis. JAMA Psychiat..

[71] van de Wiel NM (2007). The effectiveness of an experimental treatment when compared to care as usual depends on the type of care as usual. Beh. Modif..

[72] Baskin TW, Tierney SC, Minami T, Wampold BE (2003). Establishing specificity in psychotherapy: a meta-analysis of structural equivalence of placebo controls. J. Consult. Clin. Psychol..

[73] March JS (2007). The treatment for adolescents with depression study (TADS): Long-term effectiveness and safety outcomes. Arch. Gen. Psychiat..

[74] Wampold BE (2015). How important are the common factors in psychotherapy? An update. World Psychiat..

